# Efficacy and safety of temozolomide in the treatment of aggressive pituitary neuroendocrine tumours in Spain

**DOI:** 10.3389/fendo.2023.1204206

**Published:** 2023-08-31

**Authors:** Cristina Lamas, Rosa Cámara, Carmen Fajardo, Pablo Remon-Ruiz, Betina Biagetti, Fernando Guerrero-Pérez, Marta Araujo-Castro, Mireia Mora, Felicia Hanzu, Pedro Iglesias, Rogelio García-Centeno, Alfonso Soto

**Affiliations:** ^1^ Endocrinology and Nutrition Department, Complejo Hospitalario Universitario de Albacete, Albacete, Spain; ^2^ Endocrinology and Nutrition Department, Hospital Universitari i Politècnic La Fe, Valencia, Spain; ^3^ Endocrinology and Nutrition Department, Hospital Universitario de La Ribera, Alzira, Spain; ^4^ Endocrinology and Nutrition Department, Hospital Universitario Virgen del Rocío, Sevilla, Spain; ^5^ Endocrinology and Nutrition Department, Hospital Universitario Vall d’Hebron, Barcelona, Spain; ^6^ Endocrinology and Nutrition Department, Hospital de Bellvitge, L'Hospitalet de Llobregat, Spain; ^7^ Endocrinology and Nutrition Department, Hospital Universitario Ramón y Cajal, Madrid, Spain; ^8^ Endocrinology and Nutrition Department, Hospital Clínic Barcelona, Barcelona, Spain; ^9^ Endocrinology and Nutrition Department, Hospital Universitario Puerta de Hierro Majadahonda, Majadahonda, Spain; ^10^ Endocrinology and Nutrition Department, Hospital General Universitario Gregorio Marañón, Madrid, Spain

**Keywords:** temozolomide, pituitary neuroendocrine tumor, pituitary carcinoma, aggressive pituitary tumor, radiotherapy

## Abstract

Current guidelines recommend temozolomide as the first-line chemotherapy for aggressive pituitary neuroendocrine tumours. However, no clinical trials have been conducted to date and clinical experience is quite limited. We retrospectively analyzed 28 patients (9 women and 19 men), aged 46.6 + 16.9, with aggressive pituitary tumours (4 pituitary carcinomas and 24 aggressive adenomas) treated with temozolomide in 10 Spanish pituitary reference centres. Four patients had Cushing’s disease, 9 prolactinomas and 15 clinically non-functioning pituitary tumours (seven silent corticotroph, three silent somatotroph, one silent lactotroph, one silent gondotroph and three null-cell tumours). Median size at diagnosis was 10.5 cm3 (IQR 4.7-22.5), with cavernous sinus invasion in 88% and no metastases. Pre-temozolomide treatment, these data were 5.2 cm3 (IQR 1.9-12.3), 89.3% and 14.3% (2 intracranial and 2 spinal metastases). All patients had undergone surgery (1-5 surgeries), 25 (89.3%) had received radiotherapy (7 of them reirradiated) and 13(46.4%) had received cabergoline. One patient interrupted temozolomide prematurely. The remaining 27 patients received a median of 13 cycles (range 3-66) of 5 days every 28 days, with a mean initial dose of 265 ± 73 mg when administered alone and of 133 ± 15 mg when co-administered with radiotherapy. Eight patients (29.6%) had a significant reduction (>30%) in tumour volume and 14 (51.9%) attained tumour stabilization. After a median follow-up of 29 months (IQR 10-55), 8 out of these 22 showed disease progression. A longer progression-free survival was found in the five patients who received concomitant radiotherapy. Seven patients (25%) died (all of them because of tumour progression or complications of treatments) at 77 months (IQR 42-136) after diagnosis and 29 months (IQR 16-55) after the first dose of temozolomide. Adverse effects occurred in 18 patients (14 mild and 4 moderate or severe). In conclusion, temozolomide is an effective medical treatment for aggressive pitNET and pituitary carcinomas but is sometimes followed by tumour progression. Co-administration with radiotherapy may increase progression-free survival.

## Introduction

Pituitary neuroendocrine tumours (PitNETs) arise from the endocrine cells of the anterior pituitary and are usually benign in behavior. They may be found incidentally in asymptomatic patients as small and non-invasive tumours. In other cases they manifest as clinical syndromes of hormonal hypersecretion (hyperprolactinemia, acromegaly, Cushing’s disease or secondary hyperthyroidism) or with symptoms due to compression of neighbouring structures (such as headache, visual loss, hypopituitarism). Aggressive PitNETs represent only a small percentage. They have been defined as radiologically invasive tumours with unusually rapid tumour growth rate, or clinically relevant tumour growth despite optimal standard therapies (surgery, radiotherapy and conventional medical treatments). When distant metastases develop, they are classified as pituitary carcinomas (1).

Temozolomide (TMZ) is an oral alkylating agent that irreversibly damages DNA through methylation that has been approved for the treatment of gliomas (2). Its main adverse effects are asthenia, nausea, vomiting, diarrhoea and hematologic cytopenias. It is recommended by the current guidelines of the European Society of Endocrinology as the first-line chemotherapy for aggressive PitNETs and carcinomas following documented tumour growth after standard therapies (1). However, no clinical trials have been conducted so far, it is not approved for this indication by regulatory agencies and clinical experience in this context is limited, primarily arising from isolated clinical cases reports or small series of patients (3–8). Only a few lager studies have analyzed short-term and long-term response to TMZ in terms of tumour response, survival and safety (9–12). From these studies we can conclude that approximately one third of the patients treated with TMZ have a partial or complete response, one third remain stable, while one third do not respond at all. Half of the patients that initially were stable or improved, progressed in the long term. Hormonal hypersecretion usually improves in functioning tumours treated with TMZ, but time to achieve effectiveness is quite variable among treated patients. In general, there is good correlation between tumour size reduction and hormonal improvement (9–12). It has also been suggested that TMZ may had improved overall survival in patients with pituitary carcinomas (13).

All these studies have also attempted to establish predictive factors that could help to identify which patients benefit most from TMZ treatment. A lower expression of the enzyme methylguanine-DNA-methyltransferase (MGMT) and hypermethylation of its promoter in tumour tissue have been associated with a better response to TMZ in some small studies (1, 14), but these findings have not been validated in larger studies (9–12). Functioning tumours and concomitant treatment with radiotherapy have also been identified as positive predictive factors in some, but not in other studies (9–12).

Many aspects related to the efficacy and safety of TMZ in the management of patients with aggressive PitNETs and pituitary carcinomas need further investigation. When analyzing the related literature it is difficult to compare results, due to the small sample size of many studies, different criteria for the diagnosis of aggressive tumour and for defining response to treatment, patients receiving concomitant treatments (mainly radiotherapy), different duration of treatment, number of administered cycles and follow-up.

Considering this background, and taking into account that no Spanish hospital participated in the European surveys on TMZ treatment for aggressive PitNETs and pituitary carcinomas (12, 15), the aims of our study were to analyze the indications of TMZ and its efficacy and safety in the treatment of aggressive PitNETs and pituitary carcinomas in a real life setting in several Spanish university hospitals. Additionally, we sought to identify predictive factors for a positive response.

## Patients and methods

A multicentric retrospective study of patients with aggressive PitNETs and pituitary carcinomas treated with TMZ in ten Neuroendocrinology Units in Spain was conducted by the members of the Neuroendocrinology Working Area of the Spanish Society of Endocrinology and Nutrition. The diagnosis of aggressive PitNET followed the criteria proposed by the practice guidelines of the European Society of Endocrinology (1): a radiologically invasive tumour with unusually rapid tumour growth rate, or clinically relevant tumour growth despite optimal standard therapies (surgery, radiotherapy and conventional medical treatments). A pituitary carcinoma diagnosis was established in the presence of distant metastases. PitNET subtype was established based on clinical syndromes and immunohistochemistry (16). Patients were included if they had a confirmed diagnosis of functioning or non-functioning aggressive PitNET or pituitary carcinoma, had received at least a complete cycle of TMZ in the participating hospitals from 2010 to 2022, and had a minimum follow-up of six months after treatment.

Demographics, symptoms at diagnosis, biochemical parameters, imaging studies and histopathological data were obtained from the medical records, as well as previous treatments (surgeries, radiotherapy, and medical treatments) and their results. Tumour volume was estimated by the modified ellipsoidal formula (anteroposterior diameter x craniocaudal diameter x transverse diameter/2) at diagnosis, prior to TMZ treatment, at six months of TMZ treatment and at the time of best response (nadir of tumour volume). In relation to TMZ treatment, doses, number of cycles, courses of treatment, progression-free survival, overall survival and adverse events were registered. Follow-up information was collected until patient’s death or until the last visit.

Hormonal response was categorized as complete (normalization of urinary free cortisol, IGF1, prolactin or free levothyroxine, FT4), partial (>50% reduction of the above mentioned parameters, without normalization) or absent. Radiological response was considered complete when no visible tumour tissue was detected in magnetic resonance imaging (MRI) after treatment, and partial, when a reduction in tumour volume >30% was observed. Stability was defined as no change in tumour volume or a decrease <30% or an increase <20%. Progression was defined as tumour growth >20% or appearance of new metastases (9, 17).

The study was approved by the Ethics’ Committee of the Complejo Hospitalario Universitario de Albacete, and a waiver of informed consent was granted.

### Statistical analysis

For statistical analysis, categorical variables were expressed as frequency counts and percentages. Continuous variables were summarized as the mean and standard deviation when normality assumption was fulfilled and as the median and range or interquartile range (IQR) when normal distribution was not confirmed, unless otherwise specified. The Kolmogorov–Smirnov test was used to assess normality. Chi-square and Fisher’s exact test in case of categorical variables and Mann–Whitney U test and Kruskal-Wallis test for quantitative variables were used for comparisons. Kaplan–Meier survival analysis was used to analyze progression-free survival and overall survival and the log-rank test to determine whether differences in the outcomes were statistically significant. A two-tailed P value < 0.05 was considered as statistically significant in all analyses. IBM SPSS Statistics, version 28, was used for data analysis.

## Results

### Basal patients’ characteristics and therapies prior to temozolomide treatment

Twenty-eight patients (9 women and 19 men), aged 46.6 ± 16.9, from 10 Spanish Neuroendocrinology Units, treated with TMZ between 2011 and 2022, were included. Visual disturbances were present in 23 patients (82.1%), headache in 20 (71.4%) and hypopituitarism in 19 (67.9%). Four patients (14.3%) had Cushing’s disease (one of them Nelson’s syndrome), 9 (32.1%) prolactinomas and 15 (53.6%) clinically non-functioning pituitary tumours (NFPitNETs). Among the 15 NFPitNETs, immunohistochemistry (IHQ) was positive for ACTH in 7 cases, for GH in 3 cases, for prolactin in one case, for gonadotrophins in one case, and 3 tumours were negative for all pituitary hormones. IHQ showed a positive staining for p53 in 4 out of 13 (30.8%) studied tumours and the median Ki67 index was 7% (IQR 3-10). Two of the patients with a Ki67 index <3% at diagnosis, had a higher index in their second surgery (4% and 5% respectively). All pituitary carcinomas had high Ki67 index at diagnosis (range 8-20). MGMT analysis was available in only two patients: one patient had a negative immunohistochemical analysis and one patient was found to have methylation of the MGMT gene promoter.

Median tumour size at diagnosis was 10.5 cm^3^ (IQR 4.7-22.5). Cavernous sinus invasion was found in 21 patients (75%), 8 of them being Knosp grade 3-4 (all of them Knosp grade 4), suprasellar extension in 21 (75%) and inferior extension in 10 (35.7%). All patients had radiological invasion at at least one site. No patient had metastases at initial presentation.

Before TMZ treatment all patients had undergone surgery (1-5 surgeries, median 2), with an initial trassphenoidal approach in 26 (92.9%). In the second surgery a transcranial approach was used for 5 among 23 patients (21.7%). Twenty five patients (89.3%) had received radiotherapy (7 of them were reirradiated), with a median dose of 50 Gy (IQR 49.5-53). Fractionated stereotactic radiotherapy was the most common technique (21 patients, 84%), but there were two patients treated with radiosurgery, one with conventional radiotherapy and one with intensity-modulated radiation therapy. Cabergoline had been prescribed to 13 patients (46.4%), 8 prolactinomas, 4 NFPitNETs and one functioning corticotropinoma, with variable results. Two patients with Cushing syndrome were treated with ketoconazole. A therapeutic trial with somatostatin analogs was done in three patients, two prolactinomas and one silent somatotroph, with no clinical benefit. One patient had received chemotherapy with cisplatin and etoposide, without tumour response. Basal patients’ characteristics and previous treatments are summarized in **Table 1** and **Table 2**.

**Table 1:** Baseline clinical characteristics of patients and previous treatments used prior to temozolomide therapy.

VARIABLE	VALUE
Sex	9 women, 19 men
Age at diagnosis	46.6 ± 16.9
Type of tumour (functioning tumours)	
Corticotroph	11 (4)
Lactotroph	10 (9)
Somatotroph	3 (0)
Gonadotroph	1 (0)
Tyrotroph	0
Negative IHQ	3 (0)
Tumour volume (cm^3^)	10.5 (IR 4.7-22.5)
Cavernous sinus invasion	21
Cavernous sinus invasion Knosp 4	8
Previous surgical interventions (number of surgeries)	
1	5
2	14
3	6
4	2
5	1
Previous radiotherapy (Number of radiation treatments)	
1	18
2	7
Previous medical treatment	
Functioning tumours	10
Non-functioning tumours	4

Categorical variables are expressed in total numbers and numerical variables as mean ± standard deviation if normal or as median (interquartile range) if non normal. (IHQ: immunohistochemistry).

**Table 2:** Individual clinical tumour type, timeline of previous treatments and reason for prescription of temozolomide.

Patient ID	Sex	Age	Clinical tumour type	Surgeries (months from diagnosis)	Medical treatments (months from diagnosis at first prescription)	Radiotherapy (months form diagnosis)	Temozolomide (months from diagnosis at first prescription / duration of treatment, months)	Reason for temozolomide prescription
1	Woman	47	NFPitNET	4 – 32	DA (18)	43	53 / 12	Local progression
2	Man	36	PRL	62	DA (0)		78 / 40	Local progression
3	Woman	61	NFPitNET	0 – 6 – 92		10	124 / 38	Spinal metastasis
4	Man	19	NFPitNET	0 – 9 – x		0	80 / 25	Local progression
5	Woman	19	PRL	3 – 32	DA (0)	55	56 / 25	Local progression
6	Woman	54	PRL	0 – 78	DA (0) DA (88)		99 / 26	Local progression
7	Man	61	NFPitNET	1 – 3	DA (6)		4 / 18	Local progression
8	Man	14	NFPitNET	0		15	58 / 13	Local progression
9	Man	53	NFPitNET	10 – 73		75	75 / 3	Local progression
10	Woman	40	PRL	0 – 54 – 57 – 60	DA (0) SSA (62)	39 - 65	62 / 28	Local progression
11	Man	50	PRL	1 – 5 – 6	DA (0)	8	37 / 4	Local progression
12	Man	42	NFPitNET	2 – 16	DA (0) DA+SSA	28	21 / 66	Local progression
13	Man	16	PRL	0 – 71 – 73	DA (0)	77	53 / 12	Local progression
14	Woman	76	NFPitNET	0 – 22 – 30		31	40 / 11	Local progression
15	Man	34	NFPitNET	1 – 13		39 - 88	87 / 13	Local progression
16	Man	37	PRL	38 – 192	DA (144) SSA (215)	207	217 / 20	Local progression
17	Man	53	Cush	0 – 23 – 66	K (21) DA (155)	30 - 82	124 / 11	Local progression
18	Woman	65	Cush	4		3 - 7	7	Local progression
19	Woman	73	NFPitNET	12	DA (56) CT (106)	39	151 / 6	Local progression
20	Man	48	PRL	52 – 53		62	62 / 18	Intracranial metastasis
21	Man	32	NFPitNET	2 – 45 – 61 – 95 – 101		8	101	Local progression
22	Man	42	NFPitNET	0 – 6		10 - 44	62 / 14	Local progression
23	Man	59	NFPitNET	4 – 14		31 - 45	45 / 9	Local progression
24	Man	65	Cush	0 – 12		15	62 / 7	Local progression
25	Woman	55	PRL	0	DA (0) – DA (30)	30 - 74	109 / 12	Spinal metastasis
26	Man	42	Cush	1 – 22	K (29)	32	73 / 16	Local progression
27	Man	69	NoF	7 – 17		22	27 / 3	Intracranial metastasis
28	Man	44	NoF	0 – 6		47	74 / 5	Local progression

PRL, prolactinoma; NFPitNET, clinically non-functioning pituitary tumour; Cush, Cushing’s disease; DA, dopamine agonist; SSA, somatostatin analog; K, ketoconazole; CT, chemotherapy.

### Temozolomide treatment, efficacy and safety

At the beginning of TMZ treatment, median tumour volume was 5.2 cm^3^ (IQR 1.9-12.3). Cavernous invasion was found in 26 patients (92.9%), 9 of them being Knosp grade 4, suprasellar extension in 23 (82.1%) and inferior extension in 17 (60.7%). Four patients (14.3%) had developed distant metastases (2 intracranial and 2 spinal metastases). Uncontrolled hormonal hypersecretion despite other medical treatments was present in one patient with Cushing’s syndrome (one had had bilateral adrenalectomy and data were missing for the other two) and 7 out of 9 prolactinomas. The patient with Cushing’s syndrome was on concomitant treatment with the steroidogenesis inhibitor ketoconazole, and the patients with uncontrolled hyperprolactinemia received the dopamine agonist cabergoline while receiving TMZ.

TMZ treatment was started at a median of 62 months (IQR 47-96) after first diagnosis. It was given orally for 5 days in 28-day cycles in all the participating centres, with a mean initial dose of 265 ± 73 mg when administered alone and of 133 ± 15 mg when co-administered with radiotherapy (Stupp protocol, 5 patients); the dose was increased when tolerated up to 273± 85 mg, with no differences between the two groups. One patient interrupted TMZ prematurely due to severe adverse effects. The other 27 patients received a median of 13 cycles (range 3-66).

**Figure 1:**
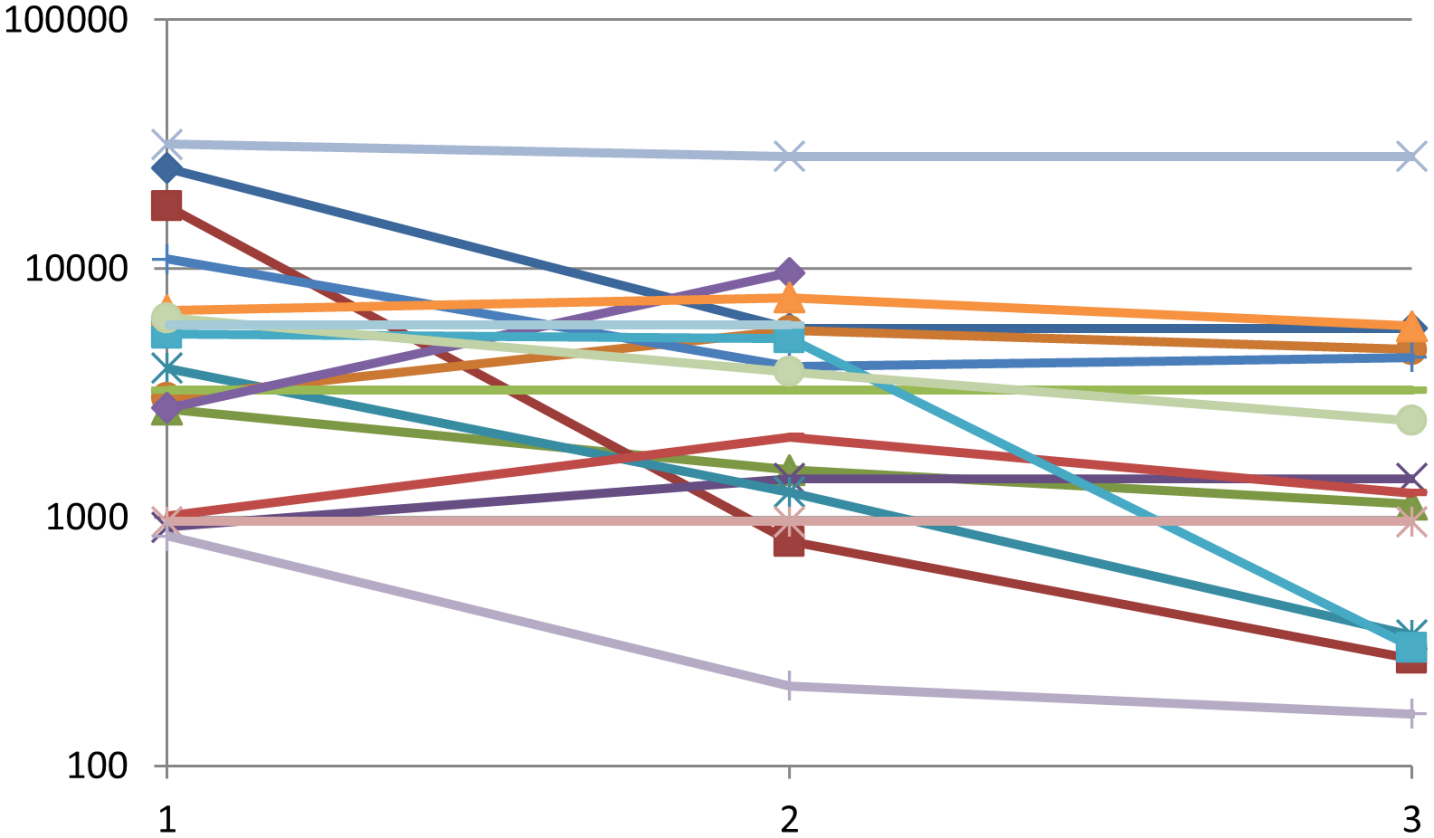
Tumour volume changes (mm3) in response to TMZ treatment, at baseline (1), at 3-6 months (2) and the best result achieved (at any time, 3). Each line represents a single patient. Nine patients were excluded from this analysis due to incomplete data for precise volume calculations.

**Figure 2:**
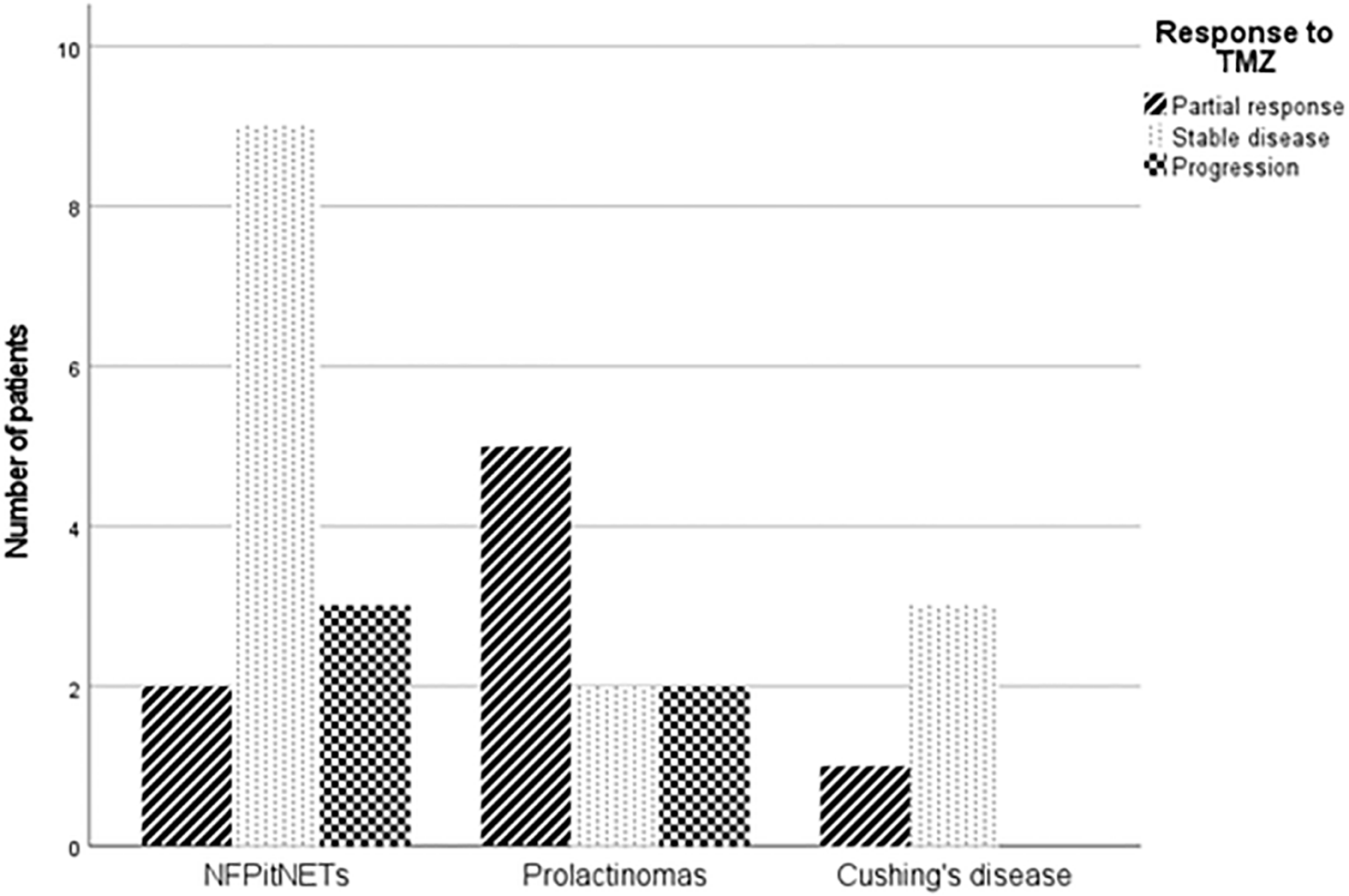
Radiological response to TMZ in the different clinical subtypes. (NFPitNETs: non-functioning pituitary tumours).

Changes in tumour volume in each individual patient are shown in **Figure 1**. No patient had a complete response. Eight patients (29.6%) had a significant reduction (>30%) in tumour volume: 3 of them had reached their maximum response in the first six months, while 5 had further reduction in tumour volume beyond that period and reached their nadir tumour volume at 11, 14, 14, 17 and 54 months respectively. Fourteen patients (51.9%) attained tumour stabilization (**Figure 2**), and 5 (18.5%) progressed. After a median follow-up of 29 months (IQR 10-55), 8 out of the 22 patients with stabilization or partial response progressed (median time from first TMZ dose to progression 16.5 months, IQR 7-27). Four patients received a new course of TMZ treatment: two of them continued their progression and were reoperated on, one had a prolonged stabilization and is still on TMZ after 66 cycles, and the forth one had just started her second TMZ course when data were collected.

**Figure 3:**
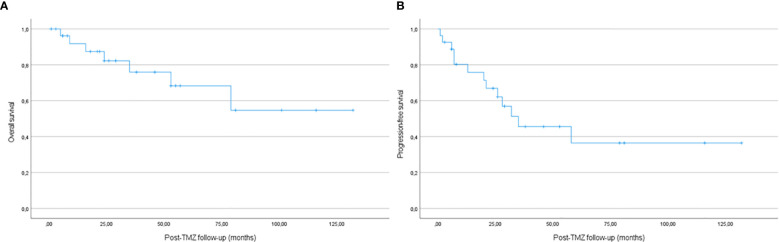
Kaplan-Meier estimates of overall survival **(A)** and progression-free survival **(B)** in the whole cohort.

Seven patients (25%) died (all of them because of tumour progression or complications of treatments) at 77 months (IQR 42-136) from diagnosis and 29 months (IQR 16-55) from TMZ first treatment. Progression-free survival and overall survival in the whole cohort are presented in **Figure 3**.

**Figure 4:**
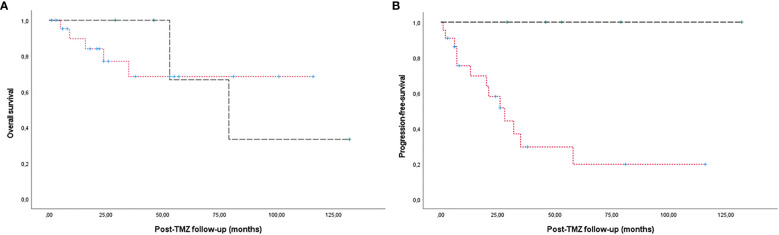
Kaplan-Meier estimates of overall survival **(A)** and progression-free survival **(B)** in patients treated with the Stupp protocol (dashed line) compared to patients who received TMZ monotherapy (dotted line). A higher progression-free survival was observed in patients treated with the Stupp protocol (p<0.05). No significant differences were found in overall survival.

When comparing patients with tumour reduction, stability or progression, we did not find significant differences in sex, age, clinical or pathological subtype, Ki67 index, p53 expression, tumour volume at diagnosis or preTMZ treatment, radiological invasion, presence of metastases, previous or concomitant radiotherapy, time from diagnosis to TMZ treatment, TMZ dose or duration of treatment or follow-up. Some of these parameters in relation to response to TMZ are shown in **Table 3**. However, progression-free survival was higher in patients treated with concomitant radiotherapy, following the Stupp protocol, compared to patients who received TMZ monotherapy (p<0.05), with no significant difference in overall survival (**Figure 4**).

**Table 3:** Characteristics of patients in relation to their tumour response to TMZ treatment.

	Partial response (n=8)	Stability (n=14)	Progression (n=5)
**Women/men**	3/5	3/11	2/3
**Age**	39.6 ± 14.8	46.5 ± 16	54 ± 17.4
**Clinical subtype** **(Cush/PRL/NFPitNET)**	1/5/2	3/2/9	0/2/3
**Ki67 index**	6.5 (4-10)	8 (4-10)	10 (3-30)
**p53 (pos/neg/NA)**	2/3/3	1/5/8	1/0/4
**ACTH IHQ**	2	7	2
**PRL IHQ**	5	2	3
**GH IHQ**	0	2	0
**Previous Radiotherapy**	8	13	3
**Concomitant Radiotherapy**	2	3	0
**Number of cycles**	14 (12-18.5)	13 (9-28)	12 (5,5-20)
**Carcinomas**	1	3	0

Categorical variables are expressed in total numbers and numerical variables as mean + standard deviation if normal or as median (interquartile range) if non normal. Differences between groups did not attain statistical significance. (Cush, Cushing’s disease; IHQ, immunohistochemistry; NFPitNET, non-functioning pituitary tumour; PRL, prolactinoma; TMZ, temozolomide. Pos, positive; neg, negative; NA, not available).

In relation to hormonal hypersecretion, the patient with uncontrolled hypercortisolism normalized his urinary cortisol, and 2 out of 7 patients with uncontrolled hyperprolactinemia normalized their prolactin levels. Hormonal response did not always parallel tumour size reduction.

Eighteen patients (64.3%) suffered adverse effects. Fourteen of them were mild and included asthenia, anorexia, mucositis, nausea, vomiting, gastrointestinal discomfort, anemia, leucopenia, thrombopenia, dizziness and adrenal insufficiency in a patient with Cushing’s disease. Four patients had more severe adverse effects: two of them had moderate gastrointestinal symptoms, associated to anemia and mucositis in one of them. A severe exanthema in one case and the development of leukemia in another led to discontinuation of treatment in two patients after 3 and 18 months respectively.

## Discussion

Clinical and epidemiological characteristics of patients treated with TMZ in Spanish university hospitals did not differ from patients from other European countries. Their mean age, 46, was very similar to that found in other series (10–12), and there was as well a clear predominance of male patients (67.9% in our series, 64% in the large European survey) (12). Corticotroph tumours, both silent and functioning, represented the most frequent lineage among these aggressive PitNETs and pituitary carcinomas, followed by lactotroph tumours, both in our cohort and all the previous multicentric studies, but we found a lower percentage of carcinomas, only 14.3%, in comparison to previously reported percentages between 19 and 32%.

Clinical practice guidelines for aggressive PitNETs recommend surgery and radiotherapy for most patients, along with standard medical treatment for functioning tumours, but they become less precise when it comes to propose subsequent treatments for tumours that progress afterwards, since scientific evidence is lacking (1, 18, 19). We found a great variability in the treatments our patients had received prior to TMZ. As expected, patients were managed in accordance with these guidelines (even if many of them had been treated before their publication): all patients had undergone surgery at some point of the evolution of their disease, received medical treatment for functioning tumours and most of them also radiotherapy. Nevertheless, there was heterogeneity in the decisions to reoperate or reirradiate or in the prescription of cabergoline or somatostatin analogs for non-functioning tumours. Consequently, the positioning of TMZ in the therapeutic algorithm was also diverse. This variability has also been found in other studies, especially if they included patients from different hospitals (9–12). Therefore, it is difficult to draw conclusions from the analysis of the published studies about the best therapeutic algorithm for patients with aggressive PitNETs and where to allocate TMZ in that algorithm.

Optimal duration of active treatment with TMZ in patients with a positive response to it is not known. The European guideline suggests a minimum of six months and our patients received a median of 13 cycles, the highest median among published reports (6-9 cycles) (9–12). Moreover, eight patients were still receiving TMZ when data were collected, with a median of 12 administered cycles (range 3-66). Longer treatments did not correlate with better responses. The reasons for discontinuation were not registered.

Less variability was found relative to the doses and therapeutic scheme (5 days in 28-days cycles) used for TMZ administration, with dose reductions when side effects occurred. Concomitant radiotherapy following the Stupp protocol was applied to 5 patients, 17,9% of our cohort, on the high limit of the reported percentages (6-15%) in other multicentric series (9–12, 15).

When it comes to analyze TMZ efficacy, we found that 29.6% of the patients had a significant reduction (>30%) in tumour volume and an additional 51.9% attained tumour stabilization. Two years after the first TMZ treatment the progression-free survival was 63.6% and the overall survival 79%. As it can be seen in **Figure 3**, most progressions occurred in the first two years after first TZM treatment. Our results are in line with previous published similar studies in terms of partial response, but with a slightly higher rate of stabilization. A first published series included 24 patients from seven European hospitals and analyzed the outcomes of 21 of them (because the other three received TMZ in combination with other treatments). A complete response was observed in two patients and a partial response in seven. One other patient attained the normalization of hormonal hypersecretion with no changes in tumour size (14). An Italian multicentric study with 31 patients treated with TMZ found tumour reduction or stabilization in 81% of the patients, but 52% regrew after a median follow-up of 43 months. Two years after treatment the progression-free survival was 47.7% and overall survival 83.9% (9).

Another multicentric study in France included 43 patients with aggressive PitNETs treated with TMZ between 2006 and 2016. After a median of 6.5 cycles, 22 patients (51.2%) were considered responders (volume or hormonal hypersecretion reduction, stable tumours are not included). Regrowth occurred in 10 of them (45.5%) after a median follow-up of 16 months. Seven were unsuccessfully retreated with TMZ and neither they responded to other drugs (chemotherapy or tyrosin-kinases inhibitors) (10). A similar multicentric and retrospective study done in Germany included 47 patients. Tumour reduction was observed in 15 patients (33%), and stability in 17 (37%) at the end of treatment, but these percentages turned down to 19.6% and 17.4% after a median follow-up of 32 months. The median progression-free survival was 23 months (11).

A survey done in 2016 by the European Society of Endocrinology among its members included 157 patients treated with TMZ and represents the largest series to date. At the end of treatment, a complete response was attained by 6% of the patients, a partial response by 31%, stability by 33% and 30% had progressed. In the long-term, two thirds of the patients had progressed at the end of follow-up (12). A second European survey on the management of aggressive pituitary tumours, published in 2022, reported data on 156 patients who received TMZ and resulted in complete response in 9.6%, partial response in 30.1%, stable disease in 28.1%, and progressive disease in 32.2% (15).

When deciding the indication of TMZ in a case of aggressive PitNET or pituitary carcinoma, it would be of interest to know the likelihood of an individual patient to respond. Larger series have identified some predictors of response: functioning tumours (10, 12), concomitant radiotherapy (12), short time interval between tumour diagnosis and TMZ initiation (10) and longer treatments (10, 20) were associated with a better response, always taking into account that association does not mean causality, since none of the studies is randomized, while other series, slightly smaller, did not find any predictor of response (9, 11). Our study stays in this side, since we couldn’t identify any predictor of response to TMZ treatment. However, we found a longer progression-free survival in patients treated with the Stupp protocol compared to patients who received TMZ monotherapy. The analysis of the expression of MGMT has yielded conflicting results: while a low expression of MGMT correlates with a positive response to TMZ in the case of glioblastomas (21), and in small series of aggressive PitNETs (14) this has not been clearly confirmed in larger cohorts of aggressive PitNETs and carcinomas (1, 10, 12). We found out MGMT immunohistochemistry and analysis of its gene promoter methylation were not yet widely available in Spanish centres in the years our patients were operated on, so we cannot broaden our knowledge relative to this controversy.

Tolerabilty of this drug was acceptable in our experience, and the reported side effects corresponded to what has been described in the literature (2, 22, 23).

Our study has obvious limitations. First of all, its multicentric nature and its retrospective design are on the base of the variability we found in clinical practice in the management of patients with aggressive pituitary tumours and carcinomas. Moreover, lack of a control group weakens any conclusion about the therapeutic effects of TMZ. However, the low prevalence of this kind of tumours makes very difficult to conduct controlled studies.

In conclusion, TMZ is an effective medical treatment for aggressive pitNET and pituitary carcinomas (81.5% tumour shrinkage or stabilization in our experience) but is sometimes followed by tumour progression (36.4%). Co-administration with radiotherapy following the Stupp protocol may increase progression-free survival. Its safety profile is acceptable, but we still face many unknowns in relation to this treatment, above all the difficulty in identifying responders prior to treatment. Future lines of investigation should clarify the best positioning of TMZ in the therapeutic algorithm, the best candidates, the ideal duration of treatment, and the possibility of a second course of TMZ in previous responders.

## Data availability statement

The raw data supporting the conclusions of this article will be made available by the authors upon reasonable request.

## Ethics statement

The study was conducted in accordance with the World Medical Association's Declaration of Helsinki and with the local legislation and institutional requirements. The study was approved by the Ethics’ Committee of the Complejo Hospitalario Universitario de Albacete, and a waiver of informed consent was granted, in line with its retrospective design.

## Author contributions

All authors contributed to conception and design of the study and collected patient’s data from their respective hospitals. CL organized the database, performed the statistical analysis and wrote the first draft of the manuscript. All authors contributed to manuscript revision, read, and approved the submitted version.
